# Peripheral blood stem cell mobilization with pegylated granulocyte colony stimulating factor in children

**DOI:** 10.1002/cnr2.1408

**Published:** 2021-07-10

**Authors:** Dhwanee Thakkar, Aseem K Tiwari, Swati Pabbi, Rohit Kapoor, Geet Aggarwal, Neha Rastogi, Satya Prakash Yadav

**Affiliations:** ^1^ Department of Pediatric Hematology Oncology and BMT Medanta The Medicity Gurgaon India; ^2^ Department of Transfusion Medicine Medanta The Medicity Gurgaon India

**Keywords:** children, mobilization, pegylated‐GCSF, peripheral blood stem cell

## Abstract

**Background and Aim:**

We report here our experience of using pegylated granulocyte colony stimulating factor (peg‐GCSF) for peripheral blood stem cell (PBSC) mobilization in children.

**Methods and results:**

A total of nine children suffering from high‐risk/relapsed solid tumors were mobilized with chemotherapy and peg‐GCSF (100 microgram/kg single dose). Mean age was 7.7 years (range 2–15 years).The mean time from peg‐GCSF administration to PBSC harvest was 9.7 days. Adequate stem cells (median dose 26.9 million/kg) could be harvested in all children by a single apheresis procedure. No major adverse events observed.

**Conclusion:**

It is feasible and safe to mobilize PBSC with peg‐GCSF in children with cancer.

## 1 INTRODUCTION

High dose chemotherapy followed by autologous hematopoietic stem cell transplant (HSCT) is a part of treatment regimens for many newly diagnosed and relapsed malignancies in children.^1^, ^2^ These autologous HSCT are most commonly performed using peripheral blood stem cells (PBSCs).^2^, ^3^, ^4^, ^5^ Various methods are known to be effective for PBSC mobilization including chemotherapy combined with granulocyte‐colony stimulating factor (GCSF) for these patients.^2^, ^3^, ^5^, ^6^ The conventional recombinant human GCSF has a short half‐life (~3.5 h) and hence needs repeated administration, which is quite painful for the child and also requires multiple hospital visits. The pegylated GCSF (Peg‐GCSF) is a longer acting version of GCSF and has a half‐life ranging from 15 to 80 h after a subcutaneous injection.^7^, ^8^, ^9^

Peg‐GCSF is a covalent conjugate between the N‐terminal methionyl residual of GCSF and mono‐methoxy polyethylene glycol (Peg) moiety. Addition of Peg moiety to GCSF increases its molecular weight and size, which results in decreased renal clearance by glomerular filtration. With this, the primary mode of elimination of Peg‐GCSF remains to be neutrophil mediated clearance.^7^, ^8^ Published studies have shown that a sustained low level of GCSF is better than short pulse‐like level to mobilize PBSC. Hence, Peg‐GCSF, might be superior to conventional GCSF in PBSC harvest in this aspect.^9^

Most of the experience with Peg‐GCSF comes from its use for prophylaxis and treatment of chemotherapy associated neutropenia in children and adults as well as for PBSC mobilization in adults.^9^, ^10^, ^11^, ^12^, ^13^, ^14^ There is paucity of literature of successful use of Peg‐GCSF and its appropriate dosing for PBSC mobilization in children.^1^, ^6^, ^13^, ^15^, ^16^, ^17^ We have attempted to summarize in Table 1 a review of published literature on use of Peg GCSF for stem cell mobilization in an all‐pediatric cohort. We report our experience of PBSC mobilization with Peg‐GCSF in nine children. It is still not a regular practice to use peg‐GCSF to mobilize stem cells in children. We through our report and review of literature, highlight that it is safe, effective, and pain‐free and could be practice changing.

**TABLE 1:** Review of literature

S.No.	Publication	Number of analyzable patients mobilized with peg GCSF	Spectrum of patients	Male: Female	Mean weight (kg)	Median weight (kg)	Mean age (Years)	Median age (Years)	Timing of use of Peg GCSF	Time of Peg GCSF administration	Dose of Peg GCSF	Capping Dose of Peg GCSF
1	Cesaro et al.^6^	36	ALL/NHL/HL 7, Solid tumors 29	1.5:1	NA	36 (14–86)	NA	10 (2.8–18.3)	Post Chemotherapy	At day +3 after chemotherapy end	100mcg/kg	6 mg
2	Fritsch et al.^1^	9	Group 2 (first time diagnosed patients) Ewing Sarcoma 3, Ependymoma 1, Neuroblastoma 1. Group 3 (patients with relapsed neoplasm) GCT 2, BNHL 1, MB 1	Group 2: M:F 4:1, Group 3: M:F 4:0	Group 2:44.6 kg, Group 3:66.5 kg	NA	13.2 (Group 2–8.6), (Group 3–19)	14	Post chemotherapy	On day 4 after chemotherapy	Group 2: median dose 200mcg/kg (100–200 mcg/kg), Group 3: median dose 195 mcg/kg (150–200 mcg/kg)	NA
3	Merlin et al.^16^	26	NB 7, Nephroblastoma 1, CNS tumor 8, HL 3, NHL 2, Sarcoma 3, others 2	NA	NA	19.3 (6–78)	NA	7.1 (1.6–16)	Hematological Steady State	More than 17 days since beginning of the last chemotherapy cycle and ANC >1 × 10^9^/L with no administration of any hematopoietic growth factor in the previous 8 days	300mcg/kg	12 mg
4	Fox et al.^13^	17	NA	11:06	NA	63.1 (39.4–101.1)	NA	17.9 (10.6–25.8)	NA	NA	NA	NA
5	Dallorso et al.^15^	22 patients (26 cycles)	NB 13, MB 2, Germinal tumor 1, Wilms' 1, NHL 1, Ewings 1, OS 3	1:1.2	NA	NA	7.5 (1–18)	7.5 (1–18)	Post Chemotherapy	At day +3 from the end of the chemotherapeutic course	100mcg/kg	6 mg
6	Carter et al.^17^	5 patients (6 occasions)	NA	NA	NA	NA	NA	NA	NA	NA	NA	NA

S.No.	Publication	Additional GCSF needed (number of patients)	Days to stem cell harvest from Peg GCSF	Days to start PBSC collection after chemotherapy	Median leukocyte peak	Median Time to TLC peak	Pre Requisite CD34 count to proceed with harvest	Mean CD34 peak	Median CD34 peak	Number of Apheresis Procedures required per patient	CD34 Stem cell dose per recipient body weight (per kg BW)
1	Cesaro et al.^6^	NA	NA	10 (6–15)	NA	NA	>20/μl	NA	143/μl (20–1988)	1 (18/29), 2 (11/29)	With first leukapheresis 8.3 (0.5–116.4) × 10^6^, with second leukapheresis 2.5 (1.2–6) × 10^6^
2	Fritsch et al.^1^	2	NA	Group 2: median 11 days (range 6–11), Group 3: median 17.5 days (8–28)	Group 2:22.9 × 10^3^cells/μl (range 19.4–44.4);Group 3:58.9 × 10^3^ cells/μl (range 7–77.7)	Group 2:13.0 days (range 7–13); Group 3:20.0 days (range 9–30)	15/μl	NA	NA	Group 2:3 (2–3); Group 3:3 (3–4)	Median‐ Group 2:24.0 × 10^6^ and Group 3:10.3 × 10^6^
3	Merlin et al.^16^	NA	4 (3–5)	NA	39.3 × 10^3^/mcl (range 9.0–113.8)	NA	20/μl at day 3 or > 10/μl after day 3	NA	NA	2 (1–4)	Median ‐ 12.4 (2.7–37.8) × 10^6^
4	Fox et al.^13^	NA	NA	NA	NA	NA	NA	165/μl (10–739)	NA	NA	NA
5	Dallorso et al.^15^	NA	5 (4–9)	NA	NA	NA	>20/μl	NA	165/μl (82.5–331)	1 (1–2)	NA
6	Carter et al.^17^	NA	NA	NA	NA	NA	>20/μl	NA	NA	NA	NA

S.No	Publication	Side effects	Success criterion	Mean success rate	Variable associated with the total number of CD34 collected	Mobilization clinically well tolerated	Patients underwent autologous HSCT	Neutrophil engraftment	Platelet engraftment	Outcome
1	Cesaro et al.^6^	None	CD34 count >20/μl	83% good mobilizers, 62% had successful collection in single leukapheresis	NA	100%	23	Day 11 (8–21)	Day13 (5–81)	22/23 alive at day 100
2	Fritsch et al.^1^	Leucocytosis	Stem cell collection: Group 2: minimum of 10 × 10^6^ CD34+ cells/kg BW; Group 3: minimum of 3 × 10^6^ CD34+ cells/kg BW	88.80%	NA	100%	NA	NA	NA	NA
3	Merlin et al.^16^	Mild fever‐ 4, nausea/anorexia −3, myalgias −1, mild bone pains −1, asymptomatic splenomegaly (2 cm) ‐2, increased plasma levels of lacticodehydrogenases ‐all, asymptomatic hyperuricemia −5, symptomatic hyperleucocytosis −0	Stem cell collection: At least 5 × 10^6^ cells/kg BW	(16/26) 60.7% (95% credibility interval: 42.0%–78.0%) achieved desired HSC harvest in one apheresis, total (22/26) 84.6% achieved desired HSC harvest in one or more apheresis	Total anthracycline dose	(20/26) 77%	21	Day 11 (9–22)	Day 15 (8–49)	NA
4	Fox et al.^13^	Mucositis, bone pain, increased hepatic transaminases. Twenty months after completion of chemotherapy, one patient on the pegfilgrastim arm developed acute leukemia with t (3; 21), +21, −8, and del 7 chromosomal abnormalities.	NA	NA	NA	NA	NA	NA	NA	NA
5	Dallorso et al.^15^	Bone pain	CD34 count >20/μl	20/26 (76.9%)	none found	(19/20) 95%	15	NA	NA	NA
6	Carter et al.^17^	NA	NA	Five (all) patients had appropriate CD34 counts for HSC Collection using a single dose of PEG‐GCSF. In 3 patients HSC collections were successful	NA	NA	NA	NA	NA	NA

Abbreviations: ALL, Acute Lymphoblastic Leukemia; BW, Body weight; CNS, Central nervous system; GCT, Germ cell tumor; HL, Hodgkin Lymphoma; MB, Medulloblastoma; NA, not available; NB, Neuroblastoma; NHL, Non Hodgkin Lymphoma; OS, Osteosarcoma.

## 2 METHODS

Nine children received Peg‐GCSF for PBSC mobilization between May 2016 and September 2020 in our unit. All these nine children were included in our analysis (none excluded). We carried out retrospective analysis of hospital records of these children. All nine children received single subcutaneous dose of Peg‐GCSF 100 μg/kg 24–48 h after completion of mobilization chemotherapy and proceeded to PBSC harvest once CD34 count was >10/μl in the peripheral blood. Stem cell collection was considered successful if we were able to collect more than or equal to 2 million/kg CD34+ stem cells. All PBSC collections were performed on COMTEC (Fresenius Kabi, Germany) apheresis machine. The product sample was taken at the end of PBSC collection from the apheresis collection bag for enumerating CD34 count in the final product. The stem cells were cryopreserved for autologous transplant. Patients were monitored for possible adverse effects of Peg‐GCSF namely bone pain, headache, injection site erythema, injection site pain, skin rash, transient hypotension, splenic enlargement, capillary leak syndrome characterized by puffiness, difficulty in breathing, and decreased urine output.^1^, ^6^, ^13^, ^15^

## 3 RESULTS

Out of 14 patients in our unit who underwent PBSC mobilization and autologous stem cell harvest, nine received chemotherapy followed by Peg‐GCSF (64%). Male: Female ratio was 3.5:1 and the mean age was 7.7 years (range 2–15 years). There were three cases of stage 4 Neuroblastoma and one each of metastatic Ewing's sarcoma, metastatic Germ cell tumor of ovary, recurrent anaplastic ependymoma, relapsed Wilms' tumor, relapsed osteosarcoma, and relapsed medulloblastoma. The data on demographic profile, diagnosis, chemotherapy received for mobilization, harvest details is shown in Table 2. For mobilization, 5/9 patients received chemotherapy, which was a part of the treatment protocol and appropriate for the underlying diagnosis, whereas 4/9 patients (relapsed osteosarcoma‐1 and neuroblastoma‐3) received cyclophosphamide‐based chemotherapy for mobilization to avoid platinum compounds proximal to harvest.

**TABLE 2:** Demographic profile, details of chemotherapy used for mobilization, CD34 count, HSC/PBSC harvest, details of engraftment of patients

S. No.	Age (Yr.)	Gender (M/F)	Weight (kg)	Diagnosis	Chemotherapy used for mobilization	Prior receipt of Platinum group of drugs (Y/N)	Prior receipt of Radiotherapy (Y/N)	Peg‐GCSF dose (mg)	GCSF before harvest (10mcg/kg)	Day of harvest from Peg‐GCSF	Day of harvest from start of chemo	CD34 count (/microliter) on the day before collection	CD34 count (/microliter) at the start of collection	CD34 count (/microliter) of collected product	Product volume (ml)	Stem cell dose (million/kg) collected	Day of Neutrophil engraftment post autologous HSCT	Day of Platelet engraftment post autologous HSCT	Duration of follow‐up post HSC harvest (months)
1	2	M	9.6	Stage 4 NB	Cyclo 2 g/m^2^	Y	N	2	—	11	12	NA	206	1756	180	32.9	9	15	16
2	3	M	12.8	Stage 4 NB	Vinc 1.5 mg/m^2^ Cyclo 2 g/m^2^	Y	N	2	—	10	11	490	NA	4002	70	28.4	8	10	6
3	3	M	11.1	Stage 4 NB	Vinc 1.5 mg/m^2^ Cyclo 2 g/m^2^	Y	N	2	one dose	10	11	16	37	1164	100	10.4	10	11	4
4	11	M	35.5	Metastatic EWS	VIDE	N	N	4	one dose	9	12	44	102	4023	120	13.5	NA	NA	4
5	10	F	24.5	Metastatic malignant GCT (ovary)	TIP	Y	N	3	—	8	14	9	NA	412	245	4.1	11	18	38
6	7	M	25.4	Recurrent Anaplastic Ependymoma	HEADSTART‐2A	N	Y	3	—	10	14	NA	200	6223	110	26.9	9	13	20
7	15	M	32	Relapsed MB	HEADSTART‐2A	N	Y	4	—	12	15	121	260	9236	210	60	10	12	6
8	13	F	28.5	Relapsed OS	Cyclo 2 g/m^2^	Y	N	3	—	10	11	10	30	703	250	6.3	12	11	5
9	5	M	17.7	Relapsed WT	Vinc 1.5 mg/m^2^ Cyclo 2 g/m^2^	Y	N	2	—	8	10	NA	330	11 090	85	53.2	8	23	8

Abbreviations: chemo, chemotherapy; Cyclo, Cyclophosphamide; EWS, Ewings sarcoma; F, Female; GCT, Germ cell tumor; M, Male; MB, Medulloblastoma; N, no; NA, not available; NB, Neuroblastoma; OS, osteosarcoma; TIP, Paclitaxel Ifosfamide Cisplatin; VIDE, Vincristine Ifosfamide Doxorubicin Etoposide; Vinc, Vincristine; WT, Wilms' tumor; Y, yes; Yr., Year.

The mean time from Peg‐GCSF administration to PBSC harvest was 9.7 days (range 8–12 days) and from start of mobilization chemotherapy to PBSC harvest was 12.2 days (range 10–15 days). Two patients required one dose of GCSF boost the day before harvest. All nine patients were harvested with single apheresis procedure. The median CD34 count at the start of harvest was 203/μl (range 30–490/μl) and median CD34 count of the final collected product was 4002/μl (range 412–11 090/μl). The median CD34 hematopoietic stem cell count collected was 26.9 million/kg (range 4.1–60 million/kg) recipient body‐weight. The mean product volume collected was 152 ml (range 70–250 ml). Four patients reported to have mild bodyache. None of the patients had any major adverse events. The median duration of follow‐up for these patient's postharvest was 6 months (range 4–38 months).

With regard to the outcome data, one patient could not reach autologous HSCT due to progression of disease. Remaining children engrafted after autologous hematopoietic stem cell infusion and all had a brisk engraftment. Neutrophil engraftment occurred at a median of 9.5 days (range 8–12 days) post autologous HSCT; platelet engraftment occurred at a median of 11.5 days (range 10–23 days) post autologous HSCT. Transplant‐related mortality was nil. Two children relapsed after autologous HSCT.

Of the remaining five patients who were mobilized with conventional GCSF, two patients (supratentorial PNET −1, relapsed medulloblastoma −1) were mobilized with GCSF and Plerixafor in hematological steady state and both required two apheresis cycles. The CD34 stem cell collected was 3.2 million/kg and 6.16million/kg body‐weight, respectively. Three patients (relapsed neuroblastoma‐1, relapsed/refractory Hodgkin's Lymphoma‐1 and relapsed sacrococcygeal teratoma‐1) underwent PBSC mobilization with chemotherapy followed by conventional GCSF. The median number of GCSF doses received was 10 (range 10–14 doses), and the mean CD34 stem cell collected was 3.1 million/kg body weight (range 3–3.2 million/kg). One of these three patients required two apheresis cycles for harvest.

## 4 DISCUSSION

The Peg‐GCSF has a longer half‐life requiring a one‐time administration as compared to conventional recombinant human GCSF, which has a short half‐life and hence requires daily administration. This makes it more tolerable and acceptable for children.^7^, ^8^, ^9^ Peg‐GCSF also provides a sustained drug level as compared to pulse like levels with GCSF, which is more effective for PBSC mobilization.^9^

There is abundant published data on the effective use of Peg‐GCSF for prophylaxis and treatment of chemotherapy‐associated neutropenia in children and adults and also about successful use of Peg‐GCSF for PBSC mobilization in adults. However, the experience on successful use of Peg‐GCSF for PBSC mobilization in children and its appropriate dosing for the same is lacking in pediatric population. From the meager published data, we can draw conclusion that it is noninferior to conventional GCSF with regard to efficacy and safety.^1^, ^6^, ^15^, ^16^ We have described here our experience of PBSC mobilization with Peg‐GCSF in nine children. Our patients received chemotherapy followed by Peg‐GCSF and we found in our cohort a fairly uniform and predictable time to CD34 peak from start of chemotherapy and from the administration of Peg‐GCSF. Also, we were able to harvest the desired CD34 stem cell dose in single harvest procedure for all our patients including the ones with relapsed malignancies who were heavily pretreated and hence deemed poor mobilizers. None of our patients had any major adverse event. All our patients who received autologous HSCT had a brisk and robust engraftment.

Fritsch et al. have reported a similar successful and safe harvest experience among their patient cohort, which comprised of first time diagnosed solid tumor patients as well as relapsed cases. Also, no other adverse events except leukocytosis had been observed in all their patients.^1^ The side effect of leukocytosis was lower in those who received Peg‐GCSF because Peg‐GCSF has a predominant neutrophil mediated elimination and hence its clearance is self‐regulating.^1^, ^7^ Dallorso et al. found a success rate of ~77% for PBSC mobilization with single dose of 100 mcg/kg of Peg‐GCSF. They also found that CD34 cell levels more than 20/μl were first observed in the peripheral blood at a median of 6 days after Peg‐GCSF administration and they remained sustained above 20/μl for a median of 6 days. This points to another appealing aspect of Peg‐GCSF that it provides a wider temporal window for planning harvest in case the peak is apparently likely to coincide with a holiday.^15^ Merlin et al. reported a success of 60% with peg‐GCSF. Target dose of 5 million/kg stem cells could be collected with a single apheresis procedure in only 16 out of 26 children despite using higher dose of peg‐GCSF 300 μg/kg.^16^ In our cohort, PBSC could be harvested successfully in 100% of patients with a single apheresis procedure. We used lower dose of peg‐GCSF 100 microgram/kg in all our patients. Lowest dose of stem cell collected in our cohort was 4 million/kg. We herein attempt to give an idea on the cost of Peg‐GCSF and conventional GCSF. Several different brands of Peg‐GCSF and conventional GCSF are available in India. Cost of Peg‐GCSF (6 mg) in India ranges from INR 3000 to 14 000 (US $41–192) and cost of GCSF (300 mcg) ranges from INR 1300 to 2800 (US $18–38) and patients mobilized with conventional GCSF usually require 7–10 doses for mobilization. This would suggest that the use of Peg‐GCSF for mobilization appears to be cost‐effective, its cost being at par with the total cost of GCSF if not less. However, we refrain from commenting on the cost‐effectiveness of one over the other due to small sample size.

Peg‐GCSF can circumvent the concerns of daily painful GCSF injections thereby improving the compliance and making the entire experience of autologous hematopoietic stem cell harvest more tolerable for children. We acknowledge the limitations of our study, it being a small and retrospective series. However, our experience highlights that it is feasible and safe to mobilize PBSC with peg‐GCSF in children with cancer and a prospective study with larger sample size should be done to validate our results. Findings of our study and review of literature could be practice changing as most pediatric transplant physicians still use conventional daily GCSF to mobilize stem cells, which is more painful for children and causes more distress and discomfort.

## CONFLICT OF INTEREST

The authors have stated explicitly that there are no conflicts of interest in connection with this article.

## AUTHORS CONTRIBUTION

All authors have contributed to this manuscript. DT wrote manuscript, AT collected data, SP‐Collected data, GA‐collected data, RK‐reviewed literature, NR‐ reviewed literature, SY‐wrote manuscript.

All authors had full access to the data in the study and take responsibility for the integrity of the data and the accuracy of the data analysis. *Conceptualization*, A.K.T., S.P.Y.; *Methodology*, D.T., R.K., G.A., S.P.Y..; *Investigation*, N.R., S.P.Y.; *Formal Analysis*, G.A., N.R., S.P.Y.; *Resources*, F.M.L.; *Writing ‐ Original Draft*, T.D.; *Writing ‐ Review & Editing*, R.K., N.R., S.P.Y.; *Visualization*, G.A., N.R., S.P.Y.; *Supervision*, N.R., S.P.Y.; *Funding Acquisition*, S.P.Y.; *Data Curation*, D.T., A.K.T., S.P., G.A., S.P.Y.; *Resources*, S.P., R.K., N.R., S.P.Y.; *Software*, S.P., R.K., S.P.Y.; *Validation*, S.P., R.K., S.P.Y.; *Project Administration*, G.A., S.P.Y.

## ETHICAL STATEMENT

Institutional approval not applicable. All patients provided consent.

## Data Availability

Data will be provided on request.
